# Developing a brief telematic cognitive behavioral therapy for the treatment of social isolation in young adults

**DOI:** 10.3389/fpsyg.2024.1433108

**Published:** 2024-08-05

**Authors:** Maria Gloria Rossetti, Cinzia Perlini, Francesca Girelli, Niccolò Zovetti, Paolo Brambilla, Cinzia Bressi, Marcella Bellani

**Affiliations:** ^1^Section of Psychiatry, Department of Neurosciences, Biomedicine and Movement Sciences, University of Verona, Verona, Italy; ^2^Unit of Psychiatry, Azienda Ospedaliera Universitaria Integrata (AOUI) Verona, Verona, Veneto, Italy; ^3^Section of Clinical Psychology, Department of Neurosciences, Biomedicine and Movement Sciences, University of Verona, Verona, Italy; ^4^Department of Neurosciences and Mental Health, Fondazione IRCCS Ca’ Granda Ospedale Maggiore Policlinico, Milan, Italy; ^5^Department of Pathophysiology and Transplantation, University of Milan, Milan, Italy

**Keywords:** cognitive behavioral therapy, telemedicine, social isolation, psychotherapy, telepsychiatry

## Abstract

**Objective:**

The present study aims to present a novel cognitive-behavioral intervention protocol focused on treating social isolation through telematic interaction, thus overcoming common barriers characteristic of face-to-face interventions.

**Methods:**

We examined current literature about face-to-face and telematic psychotherapeutic interventions for the treatment of social isolation in early adulthood. Current evidence is mixed, suggesting the need to develop novel interventions focused on patients’ cognitive functioning. Moreover, telematic interventions are promising candidates for overcoming common barriers intrinsic to the condition of social isolation.

**Results:**

The present 8-session model inspired by cognitive behavioral theoretical models and cognitive interventions currently present in the literature is thought to help socially isolated adult patients reduce clinical symptoms associated with the condition and lead to a reduction in the avoidance of social situations, leading to an improvement of the quality of life.

**Conclusion:**

We presented a telematic psychotherapeutic intervention aimed at helping adult patients suffering from social isolation who are unable to seek help from national health systems and face-to-face interventions, thus overcoming barriers intrinsic to social isolation. The present cognitive-behavioral treatment protocol has been developed in the context of a randomized clinical trial ongoing in Italy, aimed at implementing and testing the feasibility and effectiveness of multimodal digital interventions for treating social isolation.

## Introduction

Social isolation, loneliness, and social withdrawal are interrelated yet distinct phenomena that significantly impact individuals’ mental and physical well-being (Amendola et al., 2023). To understand their complexity, it’s essential to delve into their definitions, interconnections, and how they differ from each other.

Social isolation (SI) includes objective and subjective dimensions. The lack of social connections and the physical separation from other people is known as *objective* SI. Its severity is usually assessed by measuring the frequency of participation in social activities. On the other hand, *subjective/perceived* SI focuses on the emotional experience of lacking meaningful social connections and feeling disconnected from one’s social network (Cacioppo et al., 2011; Taylor et al., 2018).

Perceived SI overlaps with the construct of *loneliness,* which refers to the perceived quality of social relationships regardless of their quantity (Hawkley and Cacioppo, 2010). It is a subjective feeling of dissatisfaction with one’s social needs and the inadequacy of one’s social relationships (Beutel et al., 2017; Lam et al., 2021).

*Social withdrawal* refers to the deliberate avoidance of or disinterest in social activities and interactions (Rubin et al., 2009). A severe form of social withdrawal, observed predominantly in Japan, is the Hikikomori syndrome, defined as “a form of pathological social withdrawal or SI whose essential feature is physical isolation in one’s home” (Kato et al., 2019). Core symptoms of Hikikomori syndrome include a withdrawal period extending for at least 6 months, significant confinement at home, and considerable impairment or distress resulting from prolonged isolation (Kato et al., 2019).

SI, loneliness, and social withdrawal are complex, interconnected social dysfunctions that often represent the prodromal symptoms of several psychiatric disorders, including schizophrenia, major depressive disorder, and anxiety disorders (Bhatti and ul Haq, 2017; Porcelli et al., 2019). They may represent a transdiagnostic domain potentially independent in terms of biological underpinnings, offering prospects for targeted interventions (Porcelli et al., 2019).

Loneliness, in particular, has been associated with increased mortality risk and depressive symptoms, poorer cognitive functioning, faster cognitive decline, hypertension, and immune system dysfunction (Hawkley and Cacioppo, 2010; Taylor et al., 2018).

According to recent epidemiological studies, SI can affect up to 12% of the general population in Western countries (Röhr et al., 2022). A recent European Social Survey explored the prevalence of severe SI among the working-age population of 29 countries. The results revealed an overall prevalence of 1.7%. Italy had the second-highest prevalence in Europe at 2.17% (Amendola et al., 2023).

The data indicates that SI is a growing and increasingly widespread phenomenon in Western countries. Therefore, it is crucial to find effective treatments that address the unique clinical needs of adults experiencing SI and are well tolerated by them to improve their prognosis (Cacioppo and Cacioppo, 2014).

In clinical practice, standard psychotherapeutic interventions are typically conducted in person. Therefore, these approaches are often inadequate or only partially effective for socially withdrawn patients, for whom face-to-face interaction is a significant barrier to treatment (Gershkovich et al., 2017). Possible additional barriers to receiving treatment include geographic location, available qualified therapists, and long waiting lists.

Telemedicine can effectively address challenges in delivering mental health services to socially withdrawn individuals by offering immediate accessibility, standardization, flexibility, and affordability. Scientific literature reports that telepsychiatry and remote psychotherapeutic interventions are more adaptable, feasible, efficient, cost-efficient, and equally effective compared to traditional face-to-face treatments and psychotherapeutic interventions (Hedman et al., 2012; Gershkovich et al., 2017).

The present work aims to describe the development of a brief psychotherapeutic protocol for young adults suffering from SI to be administered via telemedicine. First, we examined the scientific literature on psychotherapeutic interventions used to treat SI-related psychological/social dysfunctions in adulthood. Based on the available evidence and our clinical experience, we developed a brief telematic Cognitive Behavioral Therapy (CBT) protocol that targets beliefs related to social impairments and clinical symptomatology associated with SI. CBT was chosen as it is considered one of the most used and reliable evidence-based psychotherapies for the treatment of psychiatric disorders and related conditions, including SI (David et al., 2018).

Our CBT protocol was developed in the context of the SOLITAIRE project, a multicenter randomized controlled trial (RCT) conducted in Italy (see Box 1).

BOX 1 **The SOLITAIRE project.**

The overarching aim of SOLITAIRE is to implement multimodal digital interventions for adolescents and young adults with SI, and their family members. Details on the project design, objectives, and methodological approach can be found at https://clinicaltrials.gov/ (ID: NCT06138301).

**Key elements of SOLITAIRE specific to our research center**

*Population*

At our research center, only young adults are enrolled. They are recruited through the psychiatry department of the local university hospital or promotional materials disseminated on social networks and in public locations of the community (e.g., universities and libraries).

*Inclusion criteria*

1. Age 18–45 years
2. Moderate-to-high levels of SI as detected by clinical evaluation and confirmed by the *Hikikomori Questionnaire - 25 items* (HQ-25) (Amendola et al., 2022)
3. Stable pharmacotherapy and symptomatology in the last 3 months
4. Absence of structured suicidal ideation
5. Not being in psychotherapy or being willing to interrupt it during the study
6. Owing a PC/tabled and an internet connection

*Informed consent and data protection*

Clinical interviews and telematic CBT are carried out through a telemedicine platform developed by the University of Milan, which consists of a patient-specialist video consultation service. The platform complies with the European privacy regulation policy (i.e., Article. 13 of EU Regulation 679/2016 for the Protection of Personal Data and Article 15 et. seq., EU Regulation 2016/679) and is built to minimize the risk of inappropriate access to the data (AWS Security standards). Eligible patients are given an informational document that describes the objectives and procedures of the study, including (among others) details on the video consultation platform used for the psychotherapy sessions and the measures implemented to ensure privacy and personal data protection. Once the informed consent form is signed, the patient is considered enrolled.

## Construction of an intervention model

### Theoretical framework

Among all the available psychotherapeutic interventions, CBT is the most extensively studied, that has been proven to be effective for the treatment of a wide variety of psychological and psychiatric conditions and has been defined as the gold standard of psychotherapies (David et al., 2018). From a theoretical perspective, CBT posits that the emotions experienced and behaviors enacted (C: consequences) by individuals stem from their interpretation of events (A: antecedents). This interpretation is what individuals tell themselves about the event or the antecedents (A), i.e., their thoughts and cognitions (B: beliefs) (Beck and Greenberg, 1974; Ellis and Bernard, 1985).

The theoretical cognitive-behavioral model of loneliness by Cacioppo and Hawkley conceptualizes loneliness as perceived SI, which can trigger implicit hypervigilance for social threats. This state of alert affects how lonely individuals perceive and interact with their social environment. Specifically, due to cognitive biases, individuals are more likely to view the social world as threatening, expect negative social interactions, and remember more negative social information. This creates a cycle where their negative expectations lead to behaviors that elicit negative responses from others, reinforcing their feelings of loneliness (self-fulfilling prophecy). Emotional consequences include heightened stress, anxiety, hostility, pessimism, low self-esteem, and diminished capacity for self-regulation. These cognition and emotional states lead to physiological and neuroendocrine changes that can lead to an increase in dysfunctional cognitions, thoughts, and biases, leading to a vicious cycle that can further impact physical health (e.g., increased blood pressure and activation of the hypothalamic–pituitary–adrenal axis, reduced social engagement, and sleep quality) (Cacioppo and Hawkley, 2009; Hawkley and Cacioppo, 2010).

Although literature lacks evidence on the efficacy of CBT protocols specifically tailored for treating SI-related psychological/social impairments, existing CBT interventions address conditions where objective/subjective SI is a core or secondary symptom. These include Social Anxiety Disorders (Wells et al., 1995), Major Depressive Disorder (Beck and Greenberg, 1974), and personality disorders (Hofmann et al., 2012), among others.

In a meta-analysis conducted by Masi et al. (2011), the authors systematically evaluated the efficacy and effectiveness of psychotherapeutic interventions aimed at reducing loneliness, defined by the authors as “the discrepancy between a person’s desired and actual social relationships” in psychiatric and non-psychiatric samples. Overall, findings highlighted that, among randomized studies, interventions targeting the modification of maladaptive social cognitions (cognitive irrational beliefs) led to greater beneficial effects compared to interventions focused on enhancing social support, social skills, and opportunities for social interaction (Masi et al., 2011).

A more recent systematic review evaluated the effectiveness of interventions to improve subjective and/or objective SI for people with mental health problems in 30 RCTs. The authors concluded that the evidence is not yet strong enough to make specific recommendations for clinical practice. However, preliminary evidence suggests that promising interventions may include cognitive restructuring for subjective SI (Ma et al., 2020).

Additional evidence from single studies shows that CBT-oriented interventions aimed at treating SI and/or loneliness in psychiatric populations (e.g., depression, anxiety disorders) led to the reduction of SI-related symptomatology (Toh et al., 2022). For example, Nagata et al. (2013) structured an in-person CBT intervention for Hikikomori patients that included both individual and group sessions. Individual psychotherapy comprised various tasks, including cognitive restructuring and emphasis on the relationship between dysfunctional thoughts and avoidance (Nagata et al., 2013). As for telematic interventions, Käll et al. (2020) proposed an 8-phase CBT aimed at reducing subjective loneliness, consisting of (i) psychoeducation on loneliness and introduction to a functional behavioral model used during treatment; (ii) identifying goals, values, and an introduction to techniques for challenging dysfunctional thoughts and beliefs; (iii) Continued work on dysfunctional thoughts and beliefs with the addition of strategies to reduce rumination; (iv) behavioral experiments, (v) behavioral activation to increase the quantity of social contact, (vi) continued behavioral activation and justification of exposure with a reduction in protective behaviors, (vii) continued behavioral activation and assessment of previous interventions; (viii) Relapse prevention (Käll et al., 2020).

### Treatment development and manualization

Based on theoretical models of SI (Hawkley and Cacioppo, 2010; Masi et al., 2011) and previous evidence, we developed a brief telematic CBT focusing on SI-related psychosocial and social dysfunctions, regardless of any psychiatric comorbidity. By targeting underlying cognitive mechanisms common to SI behaviors across various psychological conditions, we hypothesize that a trans-diagnostic approach could effectively address the core issues contributing to perceived and objective SI.

The present psychotherapeutic intervention consists of 8 weekly sessions, each lasting 45 min and with its own objective and therapeutic focus. Specifically, the overarching aim of our CBT protocol (sessions 2–7) is to treat maladaptive social cognition by reducing hypervigilance for social threats, challenging automatic thoughts, reframing perceptions of loneliness, and enhancing social cognition and engagement.

1. Session 1 - Assessment: In the first session, the therapist explores the history of the disorder, collecting significant life events and elements of the current relevant life context. The therapist also analyses the patient’s interpersonal context and reconstructs the current cognitive functioning of the disorder by identifying dysfunctional/automatic thoughts, somatic and cognitive symptoms (rumination), protective behaviors, avoidances, and emotions resulting from exposure to a feared situation. The therapist will also work on solidifying a satisfactory therapeutic alliance.

- Tools Relevant to this session: Socratic dialogue, laddering, ABC.
- Homework: Weekly diary of activities (level of activity/perception of utility, pleasure, self-efficacy in performing activities).

2. Session 2 - Sharing the disorder model: Share with the patient the previously constructed disorder model, describing, in particular, the role of identified maintenance factors (e.g., vicious cycles), aiming to (i) validate and normalize experienced symptoms and emotions, (ii) increase mentalization skills about internal states, (iii) build the therapeutic alliance, (iv) motivate treatment, (v) establish shared therapeutic goals.

- Tools Relevant to this session: ABC, psychoeducation on problematic emotions (e.g., anxiety, shame) and the disorder.
- Homework: Weekly diary of activities (level of activity/perception of utility, pleasure, self-efficacy in performing activities).

3. Session 3 - Increasing frustration tolerance and acceptance of feared events: In this session, the therapist focuses on increasing the patient’s anxiety tolerance to reduce avoidance and protective behaviors and make participation in social exposure situations more likely. This is often done by de-catastrophizing the feared scenario and its consequences.

- Tools Relevant to this session: Cognitive restructuring (de-catastrophizing scale), diaphragmatic breathing, and a safe place.
- Homework: repetition of identified alternative thought, weekly diary of activities (level of activity/perception of utility, pleasure, self-efficacy in performing activities).

4. Session 4 - Reducing self-judgment and rumination: The therapist teaches the patient a novel, non-judgmental attitude towards internal experiences. This approach will help to modify and reduce self-deprecating thoughts (double standard technique, positive reorientation, cost–benefit analysis).

- Tools Relevant to this session: cognitive restructuring (double standard technique, positive reorientation, cost–benefit analysis), compassionate mindfulness.
- Homework: repetition of identified alternative thought, weekly diary of activities (level of activity/perception of utility, pleasure, self-efficacy in performing activities).

5. Session 5–7 - Increasing the participation in social situations: In the following sessions, the therapist focuses on modifying the patient’s negative beliefs about sociality (social relationships seen as dangerous and sources of negative emotion) in favor of a representation of positivity, enrichment, sharing, closeness, and enjoyment (well-being). This will increase the range of acceptable social behaviors and promote positive cognitions about the self.

- Tools Relevant to this session: gradual exposure in imagination/*in vivo*, role-playing/modeling exercises on social situations
- Homework: gradual assignment of tasks, monitoring diary (negative emotions and thoughts before, during, and after exposure)

6. Session 8 - Vulnerability reduction: In the last session, the therapist focuses on consolidating the patient’s achieved positive changes and integrating them into a new self-model derived from comparing the new “ideal self” and “perceived self.” Moreover, the therapist identifies stressful life factors that could expose the patient to relapses, suggesting possible risk-reduction strategies.

The CBT is administered remotely by a licensed psychotherapist and a psychotherapist in training. Therapists provide telephone and email support between sessions to address patients’ inquiries and facilitate continuous engagement in therapeutic activities as needed. Patients who fail to attend the therapeutic session three times without prior notice and reasonable justification are considered drop-outs.

Once a month, psychotherapists undergo supervision sessions conducted by an experienced CBT therapist to review and optimize the therapeutic process, address challenges in treatment implementation, and provide feedback on clinical competencies.

Before and after the intervention, patients complete standardized questionnaires that measure SI-related psychological and social dysfunctions. The scores obtained from these questionnaires are then used to verify the effectiveness of the intervention.

### Expected outcomes

Based on previous literature and theoretical CBT models, we expect an overall reduction of SI and withdrawal as measured through the HQ-25 (Amendola et al., 2022; Kato et al., 2023). We operationalized the response to treatment as a post-pre-treatment reduction of ≥25% in the HQ-25 score. Additionally, we expect to observe improvements in clinical symptoms associated with SI, including, but not limited to, depression and dysfunctional and automatic thoughts in social situations. These clinical variables are measured via the Hamilton Depression Rating Scale, the Toronto Alexithymia Scale, and the Social Interaction Self-Statement Test, respectively. Due to the telematic approach used to deliver the present CBT protocol, we expect greater adherence and lower dropout rates compared to current RCTs on face-to-face CBT (Lee and Stapinski, 2012; Boettcher et al., 2013).

## Final considerations

The opportunities granted by recent advances in technology and telematic interventions have revolutionized mental health clinical services. Telematic interventions help overcome the barriers posed by traditional mental health services and face-to-face interventions. This is especially relevant for socially isolated patients. Current literature suggests that telematic interventions are equally effective and feasible when compared with standard face-to-face treatments, and, more importantly, they offer the possibility to reach patients that otherwise would not have come to the attention of the National Health Services (Gershkovich et al., 2017; Nordh et al., 2017).

Within the present study, we endeavored to develop and manualize a theory-driven cognitive-behavioral psychotherapeutic protocol designed to treat SI-related psychological disabilities in early adulthood. The present brief telematic CBT has the potential to contrast SI, improve young adult patients’ quality of life, and prevent the development of severe chronic physical, psychological, and cognitive diseases (Bhatti and ul Haq, 2017). Moreover, we believe that the present intervention has great innovation potential as it will offer psychological support to all those who are socially isolated and/or avoid showing disinterest in social interactions with others and are unable to benefit from face-to-face interventions, thus overcoming most of the obstacles present in standard interventions. Specifically, the proposed CBT intervention is intensive and brief, offering a focused approach to address SI-related psychosocial difficulties within a condensed time frame. Moreover, it integrates cognitive restructuring, behavioral activation, and psychoeducation, providing a comprehensive approach.

On the other hand, this protocol requires a high level of motivation from the patient, which may be challenging for individuals experiencing low motivation or apathy. In addition, due to the therapy’s brief nature and the possible participant’s difficulties in social interaction, there may be limited time to establish a robust therapeutic relationship between the therapist and the patient. Finally, successful implementation requires therapists’ expertise with various therapeutic tools. Looking ahead, we warrant conducting longitudinal studies to evaluate whether intervention effectiveness persists over time. Integrating new technologies, such as mobile apps or virtual reality, may further improve accessibility and engagement, particularly for patients with limited access to traditional mental health services or those with low motivation levels. Additionally, incorporating a broader range of outcome measures will enable therapists to better understand the intervention’s effectiveness on SI and related psychological dysfunctions.

## Author contributions

MGR: Conceptualization, Writing – review & editing. CP: Conceptualization, Writing – review & editing. FG: Conceptualization, Writing – review & editing. NZ: Writing – original draft, Writing – review & editing. PB: Conceptualization, Writing – review & editing. CB: Conceptualization, Writing – review & editing. MB: Conceptualization, Writing – original draft, Writing – review & editing.

## References

[ref1] Amendola S. Cerutti R. von Wyl A. (2023). Estimating the prevalence and characteristics of people in severe social isolation in 29 European countries: A secondary analysis of data from the European social survey round 9 (2018–2020). PLoS One 18:e0291341. doi: 10.1371/journal.pone.0291341, PMID: 37699030 PMC10497126

[ref2] Amendola S. Presaghi F. Teo A. R. Cerutti R. (2022). Psychometric properties of the Italian version of the 25-item hikikomori questionnaire. Int. J. Environ. Res. Public Health 19:13552. doi: 10.3390/ijerph192013552, PMID: 36294128 PMC9603413

[ref3] Beck A. T. Greenberg R. L. (1974). Coping with depression. New York, N.Y.: Institute for Rational Living, Inc.

[ref4] Beutel M. E. Klein E. M. Brähler E. Reiner I. Jünger C. Michal M. . (2017). Loneliness in the general population: prevalence, determinants and relations to mental health. BMC Psychiatry 17, 1–7. doi: 10.1186/s12888-017-1262-x 28320380 PMC5359916

[ref5] Bhatti A. B. ul Haq A. (2017). The pathophysiology of perceived social isolation: effects on health and mortality. Cureus 9:e994. doi: 10.7759/cureus.994 28382237 PMC5367921

[ref6] Boettcher J. Carlbring P. Renneberg B. Berger T. (2013). Internet-based interventions for social anxiety disorder-an overview. Verhaltenstherapie 23, 160–168. doi: 10.1159/000354747

[ref7] Cacioppo J. T. Cacioppo S. (2014). Social relationships and health: the toxic effects of perceived social isolation. Soc. Personal. Psychol. Compass 8, 58–72. doi: 10.1111/spc3.12087, PMID: 24839458 PMC4021390

[ref8] Cacioppo J. T. Hawkley L. C. (2009). Perceived social isolation and cognition. Trends Cogn. Sci. 13, 447–454. doi: 10.1016/j.tics.2009.06.005, PMID: 19726219 PMC2752489

[ref9] Cacioppo J. T. Hawkley L. C. Norman G. J. Berntson G. G. (2011). Social isolation. Ann. N. Y. Acad. Sci. 1231, 17–22. doi: 10.1111/j.1749-6632.2011.06028.x 21651565 PMC3166409

[ref10] David D. Cristea I. Hofmann S. G. (2018). Why cognitive behavioral therapy is the current gold standard of psychotherapy. Front. Psych. 9:333730. doi: 10.3389/fpsyt.2018.00004 PMC5797481 29434552

[ref11] Ellis A. Bernard M. E. (Eds.). (1985). Clinical applications of rational-emotive therapy. New York: Plenum Press.

[ref12] Gershkovich M. Herbert J. D. Forman E. M. Schumacher L. M. Fischer L. E. (2017). Internet-delivered acceptance-based cognitive-behavioral intervention for social anxiety disorder with and without therapist support: a randomized trial. Behav. Modif. 41, 583–608. doi: 10.1177/0145445517694457 28776431

[ref13] Hawkley L. C. Cacioppo J. T. (2010). Loneliness matters: A theoretical and empirical review of consequences and mechanisms. Ann. Behav. Med. 40, 218–227. doi: 10.1007/s12160-010-9210-8, PMID: 20652462 PMC3874845

[ref14] Hedman E. Ljótsson B. Lindefors N. (2012). Cognitive behavior therapy via the internet: a systematic review of applications, clinical efficacy and cost–effectiveness. Expert Rev. Pharmacoecon. Outcomes Res. 12, 745–764. doi: 10.1586/erp.12.67, PMID: 23252357

[ref15] Hofmann S. G. Asnaani A. Vonk I. J. J. Sawyer A. T. Fang A. (2012). The efficacy of cognitive behavioral therapy: a review of meta-analyses. Cogn. Ther. Res. 36, 427–440. doi: 10.1007/s10608-012-9476-1, PMID: 23459093 PMC3584580

[ref16] Käll A. Jägholm S. Hesser H. Andersson F. Mathaldi A. Norkvist B. T. . (2020). Internet-based cognitive behavior therapy for loneliness: a pilot randomized controlled trial. Behav. Ther. 51, 54–68. doi: 10.1016/j.beth.2019.05.001, PMID: 32005340

[ref17] Kato T. A. Kanba S. Teo A. R. (2019). Hikikomori: multidimensional understanding, assessment, and future international perspectives. Psychiatry Clin. Neurosci. 73, 427–440. doi: 10.1111/pcn.12895, PMID: 31148350

[ref18] Kato T. A. Suzuki Y. Horie K. Teo A. R. Sakamoto S. (2023). One month version of hikikomori Questionnaire-25 (HQ-25M): development and initial validation. Psychiatry Clin. Neurosci. 77, 188–189. doi: 10.1111/pcn.13499, PMID: 36305452

[ref19] Lam J. A. Murray E. R. Yu K. E. Ramsey M. Nguyen T. T. Mishra J. . (2021). Neurobiology of loneliness: a systematic review. Neuropsychopharmacology 46, 1873–1887. doi: 10.1038/s41386-021-01058-7, PMID: 34230607 PMC8258736

[ref20] Lee B. W. Stapinski L. A. (2012). Seeking safety on the internet: relationship between social anxiety and problematic internet use. J. Anxiety Disord. 26, 197–205. doi: 10.1016/j.janxdis.2011.11.001, PMID: 22169053

[ref21] Ma R. Mann F. Wang J. Lloyd-Evans B. Terhune J. Al-Shihabi A. . (2020). The effectiveness of interventions for reducing subjective and objective social isolation among people with mental health problems: a systematic review. Soc. Psychiatry Psychiatr. Epidemiol. 55, 839–876. doi: 10.1007/s00127-019-01800-z, PMID: 31741017 PMC7303071

[ref22] Masi C. M. Chen H.-Y. Hawkley L. C. Cacioppo J. T. (2011). A meta-analysis of interventions to reduce loneliness. Personal. Soc. Psychol. Rev. 15, 219–266. doi: 10.1177/1088868310377394, PMID: 20716644 PMC3865701

[ref23] Nagata T. Yamada H. Teo A. R. Yoshimura C. Nakajima T. Van Vliet I. (2013). Comorbid social withdrawal (hikikomori) in outpatients with social anxiety disorder: clinical characteristics and treatment response in a case series. Int. J. Soc. Psychiatry 59, 73–78. doi: 10.1177/0020764011423184 21997765

[ref24] Nordh M. Vigerland S. Öst L.-G. Ljótsson B. Mataix-Cols D. Serlachius E. . (2017). Therapist-guided internet-delivered cognitive–behavioural therapy supplemented with group exposure sessions for adolescents with social anxiety disorder: A feasibility trial. BMJ Open 7:e018345. doi: 10.1136/bmjopen-2017-018345, PMID: 29247101 PMC5735402

[ref25] Porcelli S. Van Der Wee N. van der Werff S. Aghajani M. Glennon J. C. van Heukelum S. . (2019). Social brain, social dysfunction and social withdrawal. Neurosci. Biobehav. Rev. 97, 10–33. doi: 10.1016/j.neubiorev.2018.09.012 30244163

[ref26] Röhr S. Wittmann F. Engel C. Enzenbach C. Witte A. V. Villringer A. . (2022). Social factors and the prevalence of social isolation in a population-based adult cohort. Soc. Psychiatry Psychiatr. Epidemiol. 57, 1959–1968. doi: 10.1007/s00127-021-02174-x, PMID: 34533607 PMC8445781

[ref27] Rubin K. H. Coplan R. J. Bowker J. C. (2009). Social withdrawal in childhood. Annu. Rev. Psychol. 60, 141–171. doi: 10.1146/annurev.psych.60.110707.163642, PMID: 18851686 PMC3800115

[ref28] Taylor H. O. Taylor R. J. Nguyen A. W. Chatters L. (2018). Social isolation, depression, and psychological distress among older adults. J. Aging Health 30, 229–246. doi: 10.1177/0898264316673511, PMID: 28553785 PMC5449253

[ref29] Toh G. Pearce E. Vines J. Ikhtabi S. Birken M. Pitman A. . (2022). Digital interventions for subjective and objective social isolation among individuals with mental health conditions: a scoping review. BMC Psychiatry 22:331. doi: 10.1186/s12888-022-03889-0, PMID: 35549899 PMC9098213

[ref30] Wells A. Clark D. M. Salkovskis P. Ludgate J. Hackmann A. Gelder M. (1995). Social phobia: the role of in-situation safety behaviors in maintaining anxiety and negative beliefs. Behav. Ther. 26, 153–161. doi: 10.1016/S0005-7894(05)80088-7 27816079

